# Barriers to integrating routine depression screening into community low vision rehabilitation services: a mixed methods study

**DOI:** 10.1186/s12888-020-02805-8

**Published:** 2020-08-26

**Authors:** Claire Nollett, Rebecca Bartlett, Ryan Man, Timothy Pickles, Barbara Ryan, Jennifer H. Acton

**Affiliations:** 1 Centre for Trials Research, Cardiff University, 4th Floor, Neuadd Meirionnydd, Heath Park, Cardiff, CF14 4YS UK; 2 School of Optometry and Vision Sciences, College of Biomedical and Life Sciences, Cardiff University, Maindy Road, Cardiff, CF24 4HQ UK; 3 Singapore Eye Research Institute, 20 College Road, Ngee Ann KongSi The Academia, Discovery Tower Level 6, Singapore, 169856 Singapore; 4 Centre for Trials Research, Cardiff University, 5th Floor, Neuadd Meirionnydd, Heath Park, Cardiff, CF14 4YS UK

**Keywords:** Vision impairment, Low vision, Depression, Screening, Practitioners, Training, Barriers, Optometry, Eye care

## Abstract

**Background:**

Undetected depression is common in people with low vision and depression screening has been recommended. However, depression screening is a complex procedure for which low vision practitioners need training. This study examined the integration of routine depression screening, using two questions, and referral pathways into a national low vision service in Wales at 6 months following practitioner training, and identified key barriers to implementation.

**Methods:**

This pre-post single group study employed a convergent mixed methods design to collect quantitative questionnaire and qualitative interview data on low vision practitioners’ clinical practice and perceived barriers to implementing depression screening. Forty practitioners completed questionnaires pre-, immediately post- and 6 months post-training and nine engaged in interviews 6 months post-training. Ordinal questionnaire scores were Rasch-transformed into interval-level data before linear regression analyses were performed to determine the change in scores over time and the association between perceived barriers and clinical practice. Thematic Analysis was applied to the interviews and the narrative results merged with the questionnaire findings.

**Results:**

Before training, only one third of practitioners (*n* = 15) identified depression in low vision patients, increasing to over 90% (*n* = 37) at 6 months post-training, with a corresponding increase in those using validated depression screening questions from 10% (*n* = 4) to 80% (*n* = 32). Six months post-training, practitioners reported taking significantly more action in response to suspected depression (difference in means = 2.77, 95% CI 1.93 to 3.61, *p* < 0.001) and perceived less barriers to addressing depression (difference in means = − 0.95, 95% CI − 1.32 to − 0.59, *p* < 0.001). However, the screening questions were not used consistently. Some barriers to implementation remained, including perceived patient reluctance to discuss depression, time constraints and lack of confidence in addressing depression.

**Conclusions:**

The introduction of depression screening service guidelines and training successfully increased the number of low vision practitioners identifying and addressing depression. However, standardized screening of all low vision attendees has not yet been achieved and several barriers remain. Healthcare services need to address these barriers when considering mental health screening, and further research could focus on the process from the patients’ perspective, to determine the desire for and acceptability of screening.

## Background

Low vision is defined as vision impairment that cannot be fully corrected with glasses, contact lenses or medical intervention and causes functional restriction in a person’s everyday life [1]. An estimated 129 million people have low vision world-wide [2]. In the UK, 77% of people affected are aged 65 and over [3], with the leading causes being age-related macular degeneration and glaucoma [2]. These older individuals tend to have a broader range of additional physical and mental health comorbidities compared to those without visual problems [4]. As with other chronic health conditions [5], those with low vision are at greater risk of depression than the general population [6, 7]. Depression in this population is often undetected and untreated [8, 9] as a result of the tendency for older adults to present with non-specific somatic symptoms, which may be attributed to old age or illness [10–12]; beliefs about stigma which prevent disclosure of mental health symptoms [11, 12]; or patient and practitioner beliefs that depression is a normal response to ageing [13] or chronic illness [14]. Chronic illness co-morbid with depression can lead to poorer outcomes including worse clinical symptoms and functional disability [15] and increased risk of mortality [16].

A growing recognition of the increased risk and under-detection of depression has prompted the integration of depression management into healthcare settings and reviews in primary care. In the U.S. and Canada, national guidelines recommend routine depression screening for patients with coronary heart disease [17] and diabetes [18] and in England, the Quality and Outcomes Framework incentivised General Practitioners (GPs) to screen for depression in patients with these conditions between 2006 and 2013 [19]. Such initiatives have provoked much debate about the pros and cons of routine screening. The disadvantages include longer consultations, the potential for false positives and the possible diversion of resources away from where they are needed [20]. Furthermore, if screening or case-finding instruments are used without any follow-up, they have little or no impact on depression detection and outcomes [21]. However, feeding back positive results to the clinician can improve the detection rates of depression [22], and the feedback of positive results to the patient can reduce the severity of depression symptoms by encouraging patients to take an active role in self-management [23].

In recent years, the low vision sector has identified an urgent need for depression screening to be integrated into services for people with low vision [4, 24]. In Wales, individuals can access the Low Vision Service Wales (LVSW), delivered by eye care professionals accredited as low vision practitioners. Despite a 37% prevalence of depressive symptoms in those attending the LVSW [9], our previous work indicated that in 2018, only a third of low vision practitioners aimed to identify depression and only 18% used a validated screening tool to do so [25]. The majority relied on their intuition to gain a sense as to whether a patient might be depressed. Such methods of identifying depression were shown to be less reliable than the use of a brief screening tool such as the PHQ-2 [26] by non-mental health professionals [27].

The UK’s National Institute for Health and Care Excellence (NICE) guidelines [28] recommend that health practitioners be alert to possible depression in high-risk groups. Endorsing these recommendations, the Welsh Government’s Eye Care Delivery Plan for Wales [29] asserted that all low vision practitioners should implement screening and referral pathways [29]. To support the introduction of the plan, the LVSW provided mandatory training to all practitioners in 2018.

Prior to the training, we sought to understand eye care practitioners’ views on addressing depression in people with low vision [25]. Common barriers cited were lack of knowledge about and confidence in depression identification and referral pathways, fears of ‘doing more harm than good’ and patient reluctance to discuss depression. Similar barriers were identified among Australian eye care staff and rehabilitation workers [30], who requested training in the use of a depression screening tool and clear referral pathways to improve their confidence [31]. Following training, these participants were more likely to use a screening tool and to refer depressed patients to the GP. However, time constraints, negative emotions anticipated during screening, limited referral options and patient reluctance to accept a referral remained key barriers to implementation [32]. Given the complexity of depression screening in low vision services, the aim of the study is to examine the integration of routine screening and referral pathways into a national low vision service in Wales at 6 months following practitioner training, and to identify key barriers to implementation.

## Methods

### Study design and participants

The LVSW is a national community-based service delivered in optometry practices. It supports individuals with low vision by promoting independence through the prescription of devices such as magnifiers, and by making referrals to outside agencies including voluntary organisations, social care and healthcare professionals. The service is provided by optometrists, dispensing opticians or ophthalmic medical practitioners who have undergone the Professional Certificate in Low Vision [33]. The research was granted ethical approval from the School Research Ethics Audit Committee at the School of Optometry & Vision Sciences, Cardiff University (ref. 1457/1472). All participants were given information sheets about the study prior to providing consent and all practices followed the guidelines of the Declaration of Helsinki [34].

**Fig. 1:**
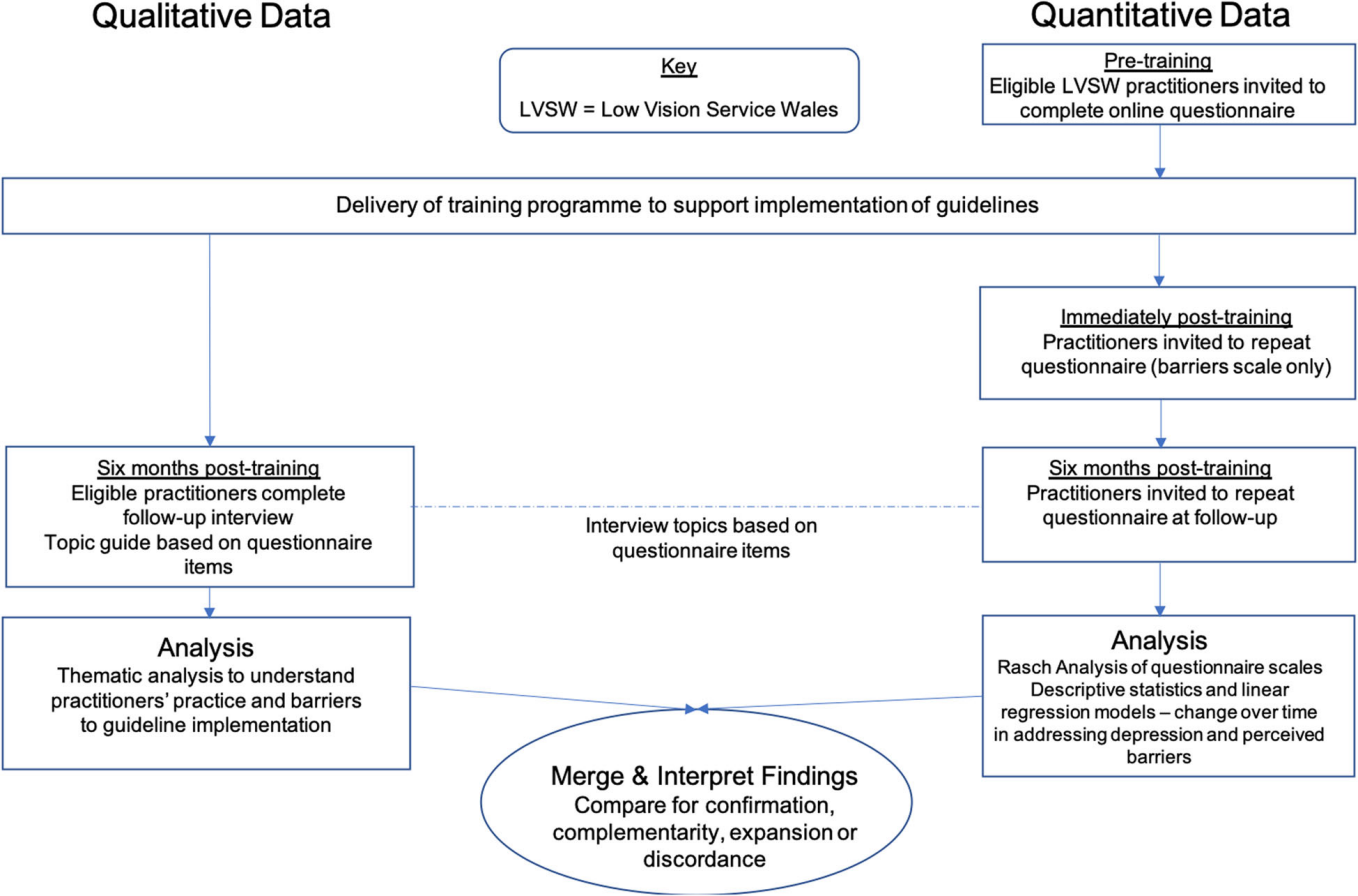
Convergent mixed methods design

The study utilised data from a broader pre-post single group study conducted by the same research team [25]. A convergent mixed methods design was employed to provide a comprehensive understanding of practice [35] (Fig. 1). Quantitative questionnaire data were collected to investigate general trends in practice and qualitative interviews provided in-depth insights into practitioners’ experiences. The two datasets were integrated to allow for comparisons between them [36]. The broader study described the practitioners’ baseline levels of perceived barriers in addressing depression, and their practice related to this prior to undertaking any training. This study reports changes directly following the LVSW training programme and 6 months later.

All practitioners were eligible (*n* = 193) excluding (*n* = 12) those who had previously completed training on depression for another research study [37] and the Clinical Lead for the service (author RB). Each practitioner was invited to take part in either the survey or the interview, to reduce burden and prevent contamination of responses.

### LVSW depression guidelines and training programme

The LVSW depression management guidelines recommend that practitioners screen all low vision patients for depression using the two Whooley questions [38], (a simplified version of the PHQ-2) [26]. The questions are:

1. During the past month, have you often been bothered by feeling down, depressed or hopeless?
2. During the past month, have you often been bothered by having little interest or pleasure in doing things?

If a patient answers ‘yes’ to either question, the practitioner should discuss this with them and offer referral to their GP for evaluation of possible depression.

The Whooley tool was chosen for its psychometric properties and ease of implementation [39], and because it is the tool recommended for use by healthcare practitioners in the NICE guidelines [28]. Both the PHQ-2 and Whooley questions showed good diagnostic performance in older people [40], however, given the latter is easier to implement (requiring no scoring), it is recommended as the preferred tool in clinical practice [40]. The combined sensitivity and specificity for the Whooley questions across six studies with older people was previously found to be 0.92 (95% CI 0.85 to 0.96) and 0.68 (95% CI 0.58 to 0.76) respectively, and for those with chronic illness, sensitivity and specificity of 0.98 (95% CI 0.85 to 0.99) and 0.86 (95% CI 0.70 to 0.94) was found respectively [39].

The mandatory training comprised of an online lecture (1 h) by a Consultant Psychiatrist, providing an overview of depression, its relevance to low vision, clinical guidelines and screening procedures. This was followed by attendance at a face-to-face workshop (1.5 h). The workshop was designed to introduce practitioners to the service guidelines and enhance their confidence in discussing depression and making a referral to the GP.

### Measures

A questionnaire was used to collect information on 1) participant demographics and employment characteristics, 2) clinical practice of identifying depression (2 questions), 3) ‘actions taken in response to suspected depression’ (8 item scale e.g. “Discuss their feelings with them”) and 4) ‘perceived barriers to working with people with depression’ (13 item scale e.g. “My limited knowledge of depression means that patients may not always receive the best management for depression”). Responses to the latter two scales were provided on a Likert scale i.e. strongly agree, agree, disagree, strongly disagree. The questionnaire was originally developed for completion by eye care professionals, refined using Rasch analysis and validated in previous research (for a full description of the questionnaire development, see Rees et al. [41]). Responses to the questionnaire were obtained pre-training (March 2018) to establish baseline levels and repeated at 6 months post-training (January 2019) to identify changes in barriers and practice following the programme (medium-term follow up). The perceived barriers scale was also administered immediately post-training (July 2018) to determine whether the training programme immediately overcame any barriers, for example, lack of knowledge of which screening tool to use. To maximise ease of completion and response rates, Online Surveys [42] was used to develop and host an online version of the questionnaire. This was iteratively tested to identify and correct errors, to ensure optimum data quality.

### Qualitative interviews

A semi-structured interview guide was developed by author CN and was based around the key topics within the questionnaire, so that the data from the two could be integrated. The guide was refined following piloting with a low vision practitioner and input from the research team and Qualitative Research Group (Centre for Trials Research, Cardiff University). Prompts were added to elicit information in the case that participants were not forthcoming. The final guide consisted of four key questions, followed by several follow-up questions and requests for examples, concerning the identification and management of depression in low vision patients and perceived barriers to addressing depression in low vision.

### Procedures

Participants who had completed a pre-training qualitative interview as part of the wider study [25] (*N* = 12) were invited by email to take part in a follow-up interview, 6 months after the training. Participants were selected for the interviews using a maximum variation strategy with the purpose of eliciting the views of practitioners with a range of characteristics and experience [43]. Interviews were conducted by CN, a female psychology graduate and experienced mental health researcher interested in the association between vision loss and mental health. CN was independent of the LVSW and unknown to the practitioners. Interviews were undertaken either face-to-face in the practitioner’s place of work or in a university office, or by telephone, and lasted between 30 and 56 min. Field notes and a reflexive journal were kept throughout the interviewing and analysis process.

All other eligible practitioners were invited by the LVSW Clinical Lead to take part in the online questionnaire (*N* = 167). Practitioners were emailed a link along with an introduction to the study and the Participant Information Sheet. They were informed that they could complete the questionnaire solely as a pre-training reflection task (a standard aspect of LVSW re-accreditation training) or could check a box to indicate their consent for their answers to be used for research purposes. Practitioners were asked to provide a pseudonym to anonymise their responses, which could then be linked across the three time points. Anonymity was considered important to reduce perceived pressure to consent and to maximise the likelihood of honest responses. Further links to the questionnaire were sent immediately following the face-to-face training and 6 months post-training. At each time point, a reminder email was sent to maximise participation.

### Analysis

#### Psychometric assessment of questionnaire scales

Rasch analysis was used to assess the psychometric properties of the ‘actions taken in response to suspected depression’ and ‘perceived barriers’ scales using the Andrich rating scale model [44] with Winsteps software (version 3.92.1), Chicago, Illinois, USA. Since participant scores are ordered counts, uneven and non-linear, a simple summation of raw scores and/or reporting of proportions as is traditionally carried out is demonstrably flawed [45]. Rasch analysis, a form of Item Response Theory (IRT), was undertaken to transform these ordered responses to estimates of interval measures (expressed in log of the odds units, or logits). Once the data fit the Rasch model, person measures in logits were extracted for further analysis. During Rasch analysis, responses were recoded so that higher scores indicated greater willingness to act in response to perceived depression in low vision patients (“action taken” scale) and greater perceived barriers in working with low vision patients with depression (“perceived barriers” scale), respectively. To generate valid pre-post person measures, data were stacked and anchored to item calibrations at baseline [46]. DIF was assessed for age (median split < 44 years vs. ≥44 years), gender, data collection time points (baseline, post-training, follow-up) and whether the practitioner had previously received training in the identification and management of depression.

#### Statistical analysis

STATA Version 13 (StataCorp LLC, TX, USA) was used to analyse the questionnaire data. Participants who completed the questionnaires at all three time points constituted the final sample and were included in the analysis. Descriptive statistics were used to describe their demographic and work characteristics and their reported practice around the identification of depression. Categorical variables were summarised as numbers and percentages whilst continuous variables were presented as medians with interquartile ranges.

Two multi-level linear regression analyses (multiple timepoint observations clustered within person) were conducted to determine the difference in scores over time for the ‘action taken in response to depression’ scale and the ‘perceived barriers’ scale (reference category: pre-training). A further multi-level linear regression was performed to examine the relationship between perceived barriers and action taken. No other variables were entered into the model. The two-tailed *p*-values and 95% confidence intervals of each analysis are presented with *p*-value < 0.05 considered statistically significant.

#### Qualitative analysis

A professional company transcribed the audio-recorded interviews verbatim. The interviewer checked the transcriptions for accuracy before analysing the data using a codebook approach to Thematic Analysis [47]. The first step was familiarisation with the data through repeatedly reading each transcript. Initial codes were then generated inductively from the data and recorded on each transcript, before being transferred to electronic copies stored in NVivo (Version 11) where they were organised (eg grouped, renamed or divided). They were subsequently grouped under three themes, mirroring three sections of the questionnaire: identification of depression, action taken in response to suspected depression and perceived barriers to working with people with depression. This facilitated integration of the two sets of data. Sub-themes were generated inductively from the data.

To ensure rigour, the codes and sub-themes were checked against the interview transcripts, the interviewer’s reflexive journal and discussed with the research team to ensure they remained true to the original data. They were then written into narratives evidenced by data extracts. The analysis was approached from a realist perspective and codes were developed at a semantic level, by examining the surface meaning of the data [48].

#### Mixed methods integration and analysis

In a convergent design, integration is a process of combining the quantitative and qualitative data to enhance understanding and validate the results [36]. Integration occurred both at the methods level, through basing interview questions on the questionnaire sections, and at the results level, through *merging* [36, 49] questionnaire and interview findings. When merging the two sets of results, four possible outcomes were considered [50]: 1) *Confirmation*, when the quantitative and qualitative findings lead to the same interpretation 2) *Complementarity*, when the two sets of data show different, non-conflicting conclusions 3) *Expansion,* when the datasets provide a central overlapping theme and a broader non-overlapping interpretation 4) *Discordance*, when the two datasets lead to conflicting interpretations.

## Results

The psychometric assessment of the two questionnaire scales are reported first, followed by the main results centred around the four sections of the questionnaire. For each section, we present the questionnaire data, followed by the corresponding interview data and mixed method outcomes where applicable.

### Psychometric properties of questionnaire scales

Initially, both scales displayed poor fit to the Rasch model, with suboptimal precision, evidence of multidimensionality (> 1 construct being assessed) and DIF, as well as misfitting items. After iterative removal of misfitting items and those displaying DIF (2 items from the action in practice scale and 3 items from the barriers scale), the two questionnaire scales displayed adequate psychometric properties with ordered response thresholds, good precision (able to distinguish at least 3 levels of participant ability), no misfitting items or DIF, and importantly, no evidence of multidimensionality. The difference between item difficulty and person ability for both scales was minimal, indicating that the items were suitably targeted to the participant population. Person measures (in interval level log-odds units [logits]) were then exported for use in subsequent parametric testing. The mean person measures (±SD) at baseline were − 1.38 (2.30) logits and − 0.47 (1.37) logits for the “action taken” and “perceived barriers” scale respectively, while the mean scores at 6 months follow-up were 1.39 (2.30) logits and − 1.42 (1.65) logits respectively i.e. there was more action and less perceived barriers at 6 months (Table 3).

### Demographic and employment characteristics

A total of 40 participants completed all three questionnaires, with their results utilized in the pre-post quantitative analyses. Their baseline demographic and employment characteristics of these participants are presented in Table 1.

**Table 1:** Summary of questionnaire and interview participants’ demographic and employment characteristics (collected pre-training)

Characteristic	Questionnaire ***N*** = 40		Interview ***N*** = 9	
Gender				
Male	N (%)	14 (35.0)	N	5
Female		26 (65.0)		4
Professional Background				
Optometrist/Ophthalmic medical practitioner	N (%)	37 (92.5)	N	7
Dispensing optician		3 (7.5)		2
Time employed as LVSW practitioner (years)	Median (IQR)	10.0 (6.5–12.0)	Range	1–12
Average number of people with low vision seen each month	Median (IQR)	5.0 (4.0–12.0)	Range	1–60
Average time spent with patient with low vision (mins)				
Less than 10	N (%)	0 (0.0)	N	0
11–20		0 (0.0)		0
21–30		3 (7.5)		0
31–40		9 (22.5)		1
41–50		16 (40.0)		3
51–60		10 (25.0)		5
More than 60		2 (5.0)		0

Nine of the 12 practitioners approached completed a qualitative interview: of the remaining three, one was on maternity leave and two agreed to a second interview but could not schedule one during the data collection period. Interview participants’ characteristics are presented in Table 1.

### Identification of depression

The number of practitioners who indicated on the questionnaire that they aimed to identify depression increased from 15 (37.5%) at pre-training to 37 (92.5%) at 6 months post-training, with 35 (87.5%) reportedly using the two Whooley questions. Whilst only 4 (10%) reported using any screening questions more than ‘rarely’ prior to the training, 32 (80%) reported doing so 6 months post-training (Table 2). However, only 13 (32.5%) reported doing so ‘always/almost always’, as per the service guidelines.

**Table 2:** Frequency of using a screening tool to identify depression before training and 6 months post-training

	Pre-training		Six months post-training	
	*n*	*%*	*n*	*%*
Never/rarely	36	90	8	20
Less than half the time	1	2.5	7	17.5
More than half the time	3	7.5	12	30
Always/Almost always	0	0	13	32.5

Amongst the interview participants, the most commonly reported outcome of the training programme was an increased awareness of the prevalence of depressive symptoms in people with low vision. As a result, some practitioners had started to enquire about how patients were feeling.

*“I think I try to be more aware and look at the patient and question them about how they feel.”* P05

#### Practitioners adapt the guidelines

The service guidelines require that all patients are screened. Eight of the practitioners acknowledged this guideline but had mixed views about its utility. They shared two variations to its implementation. Firstly, the majority did not screen every patient, reporting that they considered it inappropriate to ask the two questions of people who they considered to seem well or healthy, which they judged from behavioural cues and conversation.

“*…*. *I* *would say that, er, I probably ask that (the screening tool) to about half of them …*. *if they seem to be, um, absolutely on top of the moon, and bubbly, positive, and, um, everything I just have a feeling that there isn’t anything to it. There’s no point in asking it.”* P08

Secondly, only one practitioner asks the two questions exactly as worded. The remainder revealed that they change the wording or ask similar questions instead, mainly to avoid using the words ‘depressed’ or ‘hopeless’ which are viewed as ‘loaded’ or ‘taboo’. Some practitioners fear that by talking about depression they could “*make matters worse*” (P05) or stir up difficult feelings.

*“I try and avoid using it (depression) directly, especially too early on in the conversation. I think it's a very taboo word these days isn't it? … .I try and just talk about things like the sort of moods and feelings and things like this, rather than depression itself.”* P03

*“ … .I’m worried about bringing up the big depression word with patients, I’m worried about causing more trouble.”* P07

In addition, practitioners perceive that asking the questions word-for-word appears unnatural, robotic or like a ‘tick box’ exercise, and prevents the smooth integration of depression screening into the assessment. Instead, they ask the questions in their own way, perceiving that conveying the gist of the questions is sufficient.

*“I don’t word it exactly as heard, I think it might sound a bit robotic if I did … … erm, but I try to make sure that I, I get the main focus or point of the sentence or question across.”* P07

#### Appears acceptable to patients

Most practitioners had found a way to introduce questions about emotional health when they felt it was appropriate and usually incorporated this into the typical history and symptom taking. Seven explained that despite their concerns, questions about mood/depression seemed to be acceptable to patients. Moreover, in some cases, patients who were feeling depressed, expressed relief and gratitude at being encouraged to talk about it. This reinforced the need for discussions with future patients.

*“ … d’you know, I’ve not had one single negative response?.....But there's been a lot of people who’ve just been really grateful”* P11

#### Mixed method outcome

*Confirmation.* The two sets of results confirmed that more practitioners aimed to identify depression after training and were using the screening questions to do so, but not at every assessment. The interviews revealed that the majority only use the screening questions when they feel it is appropriate, and they adapt the wording of the questions at their discretion.

### Action taken in response to suspected depression

The results demonstrate a statistically significant increase in practitioners’ total score on the ‘action taken in response to suspected depression’ questionnaire scale (Table 3), indicating that practitioners reported taking more action than before training. At both time points, practitioners were most likely to discuss the patients’ feelings with them, discuss a GP referral and refer to the GP. They were least likely to refer patients to a mental health service. For the full range of responses see Additional file 1 - Supplementary Figure 1.

**Table 3:** Mean scores for questionnaire scales & linear regression to determine difference in score over time

Scale	Timepoint	Mean	SD	Linear Regression		
				Difference in means	95% CI	***p***-value
Action taken	Pre-training	−1.382	2.3073	Reference category		
	Follow-up	1.386	2.3030	2.768	1.925 to 3.611	< 0.001
Log likelihood = − 177.608; Wald χ^2^ = 41.42, *p* < 0.001						
Perceived barriers	Pre-training	−0.474	1.3793	Reference category		
	Post-training	−1.121	1.4637	−0.647	−1.012 to − 0.282	0.001
	Follow-up	−1.424	1.6457	−0.950	−1.315 to − 0.585	< 0.001
Log likelihood = − 188.596; Wald χ^2^ = 27.15, p < 0.001						

The linear regression to examine the relationship between barriers and action taken revealed that practitioners who perceived more barriers took less action in response to suspected depression (beta coefficient = − 0.805, 95% CI − 1.133 to − 0.477, *p* < 0.001).

In responding to possible depression, most practitioners who were interviewed considered that the usual activities undertaken in a low vision assessment could be enough to have a positive impact on mood. For example, improving visual function through rehabilitation can enable engagement in valued activities and signposting to social care organisations can facilitate additional support.

*“..now you think how, um, people's loss of vision impacts on sort of … .their mood if you like. Um, and then it also makes you realise what a difference you can make by getting them to see better … .”* P04

#### Few GP referrals made

In addition to improving visual function, discussing a GP referral was the most common action taken in response to suspected depression; however, its implementation differed among practitioners. Some solely advised the patient to visit the GP, whilst others discussed a referral with the patient and indicated they were “*happy to write a letter”* (P12) if the patient agreed. However, following through with a referral was uncommon, sometimes because the patient was already under the care of the GP, but more often because the patient reportedly declined the referral.

*“They're usually fairly unmotivated … ..to get themselves sorted”* P03

*“It's so hard sometimes, just to get people to accept help”* P04

Practitioners reported being more likely to make a referral when they suspected a patient was seriously depressed or suicidal.

*“If I thought they were really totally depressed, and I was worried for them, then I would contact the GP, and make a referral just to say, I've seen this patient, I think she has a lot of difficulties, in coping, and I would like your opinion.”* P05

#### Mixed methods outcome

*Confirmation & Expansion.* The questionnaire outcomes indicated an increase in action taken, with discussing feelings and a GP referral being more likely than mental health referrals. The interview data confirmed that discussions about GP referrals commonly occurred and additional information about the barriers to referral was gained. Referral to social care and other activities to promote visual function were viewed as having the potential to influence mood, which was not measured on the questionnaire.

### Perceived barriers to working with people with depression

The total scores on the ‘perceived barriers’ questionnaire scale decreased significantly between pre- and post-training, with a further significant decrease at 6 months (Table 3.) The most commonly endorsed barrier at each of the time points was the patients’ reluctance to discuss how they feel. Supervisor support, workplace environment and a need to protect oneself from patients’ emotional problems were the least endorsed barriers. For the full range of responses see Additional file 2 - Supplementary Figure 2.

#### Patients themselves are a barrier

In the interviews, the most commonly perceived barrier to addressing depression in patients with low vision was ‘the patient themselves’. Patients were perceived as being reluctant to talk about depression, due to the sensitive nature of the topic and a view that it is a sign of weakness.

*“ … a lot of people that come in are very, very brave, stiff upper lip and all that, and they don’t share much with you.”* P04

However, this was a motivating factor for one participant to ask everyone the screening questions, rather than rely on the patient to initiate a discussion about mood:

“*I do make a point (of asking everyone) because I’m aware that so many people won't bring it up themselves for reasons, for a million reasons, I suppose, if they were either ashamed or just trying to deny it, or didn’t feel like it was my place to discuss it with them … .so you have to do it for them.*” P11

The perception that individuals can mask depression is seen as a barrier to identification. Practitioners reported being unsure how to negotiate situations in which the patient reported being fine, but they suspected otherwise. This is particularly an issue for those who do not ask the two questions, but rather rely on their intuition or indirect questioning.

*“Barriers? … I think sometimes, erm, you know if someone’s depressed and they put on a sort of façade of a bubbly, bon homie, um … and maybe I’m not as good at identifying that.. … probably I should ask everybody that (screening) question ...”* P08

Most practitioners perceive that, in general, patients are reluctant to accept help for emotional difficulties which then makes instigating a referral to the GP difficult. It was acknowledged that simply advising the patient to visit their doctor was not enough to lead to any action, but that a formal referral cannot be made without the patient’s consent.

*“Oh yeah, yeah, no problem if someone’s happy for me to refer them, no problem at all. Erm, I think, I mean the only barrier is the patient themselves, I guess, if they decline then I can’t really deny that”* P07

Discussing support options and gaining consent was an area in which practitioners wanted further training.

#### Service barriers

A few practitioners reported the lack of time is a barrier and *“some things can take more precedence”* (P04) over depression screening in low vision assessments. They explained that when patients openly discuss their mental health, appointments can take longer than usual and impact on clinic management. However, others felt they did have enough time, and certainly more than GPs, and were therefore well placed to ask about mental health. Long waiting times for mental health services and perceived lack of action by GPs were seen as barriers to obtaining support for patients, over which they had no control.

*“ … it’s things that are outside my control perhaps. I mean I know obviously we all hear about the NHS and the issues and I know about mental health waiting times and I know about, erm, lack of resource funding etc. I, I can’t think of, of anything to fix that.”* P07

*“I have had two patients, um, recently that I’ve seen now for the second time … .where I’ve made a referral for depression. Um, and I’ve said, “Oh,” you know, “what happened after that, then?” And both of them said, “Nothing.”* P11

#### Practitioner lack of confidence

For some practitioners, lack of confidence was less of a barrier than at pre-training, because they felt more certain about how to identify and discuss depression.

*“I think the barriers that I had before was that I didn’t know the right way to go about asking, and now I feel happy that I have the two questions, um, so I know, I know how to approach it.”* P12

However, confidence remained a barrier to addressing depression for those who do not feel comfortable asking the two questions and or in gaining consent for a referral to the GP.

*“Um, how do I convince them that it would probably be better if I did the referral, rather than just relying on them to go (to the GP)”* P03

#### Mixed methods outcome

*Confirmation*. The two datasets confirmed that overall barriers to working with patients with depression decreased, and that the key remaining barrier was perceived patient reluctance to talk about depression or to accept help.

## Discussion

This study examined the integration of depression screening and referral pathways into the LVSW. Importantly, we found that over 90% of practitioners aimed to identify depression in their patients following training, compared with only one third beforehand. Most reported using the recommended Whooley questions to do so. However, only a minority used the screening questions with all patients, despite the specification to do so in the service guidelines, based on their perception that it is inappropriate to screen people who seem healthy and well. In addition, the majority of practitioners did not consistently ask the questions as phrased. Instead, practitioners modified the wording because of a fear that direct conversations about depression may make matters worse, and to prevent screening from appearing like a ‘tick box’ exercise. Despite practitioners’ concerns about addressing depression, the majority perceived that patients accept being questioned about their mood and some even showed relief or gratitude.

Practitioners took significantly more action to address possible depression following the training. Those who perceived fewer barriers were more likely to take action such as discussing the patients’ feelings with them, and offering and making a GP referral. However, interview participants revealed that discussions about referral are not straightforward and gaining consent can be difficult due to their patients’ general reluctance to accept help. Referrals for perceived cases of severe depression or suicidal ideation were more likely than for more moderate cases. A further course of action taken concerned improving visual function, which some practitioners believed to be the cause of, and therefore the solution to, depression.

Overall perceived barriers to addressing depression reduced significantly. Although practitioners gained in confidence, the key remaining barrier was perceived to be ‘the patient themselves’. Specifically, patients’ apparent reluctance to discuss their feelings and to accept support. Some practitioners believed people can hide depression well, making it difficult to identify. Failure to screen every patient may compound this issue. Other barriers cited were limited appointment times leading to prioritising eye related matters, and issues beyond the practitioners’ control such as long waiting times for mental health services and lack of treatments offered by the GP.

Our findings align with previous work, in which brief training programmes for Australian eye health and rehabilitation workers reduced their perceived barriers and increased their likelihood of responding to depression [32, 41]. Staff were less likely to report that lack of skills, training and ability to identify depression were barriers at post- compared to pre-training, but as with our findings, time constraints and patient unwillingness to accept a referral remained key concerns. The Australian studies [32, 41] assessed the impact of training at only one timepoint (on completion of the training), and utilised only quantitative data. As suggested by the authors of the two studies, we have conducted a 6 month follow-up evaluation and used interviews to supplement questionnaire measures in the present study, thereby extending the evidence base.

The wider literature on depression screening in general healthcare settings similarly identifies considerable barriers. Low rates of screening have been reported across primary care [51], physiotherapy [52] and hospital-based stroke services [53], even when staff have received specific training or are incentivised. Consistent with our findings, one of the main barriers reported by primary care practitioners is the desire to avoid a ‘tick box exercise’ and their subsequent struggle to align the use of a screening tool with a patient-centred approach [14, 54, 55]. Practitioners across healthcare settings reported: using a standardized test only with people they suspected were depressed [53]; avoiding asking screening questions if they were familiar with the patient [55]; and adapting the questions to suit their consultation style [54–56]. Furthermore, use of the Whooley questions in routine practice has been shown to miss cases of depression, which may be due to variations in how the questions are asked [56]. In addition, nurses expressed discomfort with asking the two questions, suggesting it was like ‘opening a can of worms’ which was difficult to address in the allotted assessment time [54, 55].

Concerns over patient reticence to discuss depression also permeate the wider literature [14, 54, 55]. For example, patients with long term conditions screened for depression in primary care did not understand why they were being screened which led to some giving defensive answers [55]. In maternity services, patients were less likely to disclose depression when it was felt that staff were unconfident or ‘going through the motions’ [57]. Conversely, patients with low vision being screened for depression responded positively to the process [58].

### Implications for practice

The complexity of integrating two depression screening questions into a low vision, or any healthcare/rehabilitation setting should not be under-estimated. Alderson et al. [55] recommended patients, professionals and the service should be fully prepared. Whilst our guidelines and training proved helpful, future training should have a stronger focus on how to integrate the screening into the assessment in a patient-centred way. This might be achieved through role play, ideally with professional actors taking the part of patients. Enhanced knowledge of treatment options and outcomes offered by the GP may increase confidence in discussing and gaining consent for a GP referral.

Patients could be prepared either by careful introduction and explanation of the questions during the assessment, or via information sent out prior to their appointment. This may help reduce feelings of shame and enable patients to discuss depression [19]. Finally, service preparation may include the consideration of extended assessment times, adding the screening questions to assessment schedules and providing ongoing support for practitioners.

Patient outcomes also need to be considered. In this study, we did not record the impact of screening and/or referral on depression. The evidence as to whether simply referring to the GP leads to improvements in depression for people with vision impairment is mixed [8, 37]. Identifying positive cases may be of little benefit if there are no effective assessment or treatment services available [39]. Instead, it may be more prudent to focus on interventions for the prevention of depression in this high risk group [59]. In addition, screening in primary care may provide further opportunities for detection and treatment.

### Strengths and limitations

Unlike many previous studies in low vision, we conducted a medium-term follow-up. Therefore, participants were able to report changes incorporated into their practice, rather than their intention to change. Use of a mixed methods design provided a comprehensive understanding of practice and overcame the potential limitations of using either quantitative or qualitative methods alone. The two datasets confirmed each other, suggesting valid and credible results, and the interview data explained some of the questionnaire findings. The questionnaire data were transformed from ordinal to interval-level responses using Rasch analysis and the reliability of the questionnaires was demonstrated. Anonymous completion of the questionnaires may have facilitated more honest answers than otherwise expected.

The study is limited by the absence of a control group and we cannot be sure that any changes which occurred were due solely to the introduction of the guidelines and training programme. A valid control group was not feasible for this study, as all LVSW practitioners underwent mandatory training. Despite this, only around a quarter completed a questionnaire at all three time points and were included in the study. We do not have any information on those who were not included, therefore there may be a risk that the completers differed from the non-completers. However, our rich qualitative dataset supported the questionnaire results, suggesting our findings are representative of practitioner views. The results from the nine participants who were interviewed at follow-up may be subject to selection bias inherent in agreeing to take part and more interviews would have enabled greater data saturation. The study relied on self-report data to assess change in practice. Despite anonymous completion, this method of data collection is still open to bias. Further studies would benefit from using routine data or observing clinical practice.

## Conclusions

The introduction of depression screening guidelines and training successfully increased the number of practitioners identifying and addressing depression in individuals attending the low vision service. However, standardized screening of all attendees to the service using the two Whooley questions has not yet been achieved and several practitioner, patient and service barriers remain. Further research could focus on the process from the patients’ perspective, to determine the desire for and acceptability of screening. More work is needed to understand the impact of screening and referral on patient outcomes.

## Supplementary information

**Additional file 1: **Responses to ‘Action taken in responses to suspected depression’ Scale. **Supplementary Figure 1.** Indicates the responses to the ‘Action taken’ scale at pre-training and 6 months post-training.

**Additional file 2: **Responses to ‘Perceived barriers’ Scale. **Supplementary Figure 2.** Indicates the responses to the ‘Perceived barrier’ scale at pre-training and 6 months post-training.

## Data Availability

The datasets used and analysed during the current study are available from the corresponding author on reasonable request.

## Abbreviations

GP: General Practitioner
LVSW: Low Vision Service Wales
NICE: National Institute for Health and Care Excellence
PHQ-2: Patient Health Questionnaire
