# Supplementary material for: Barriers to integrating routine depression screening into community low vision rehabilitation services: a mixed methods study
Source: BMC Psychiatry. 2020 Aug 26;20:419. doi: 10.1186/s12888-020-02805-8 (PMC7448511; doi:10.1186/s12888-020-02805-8)
Supplement: Supplementary file 1 — Additional file 1: Responses to ‘Action taken in responses to suspected depression’ Scale. Supplementary Figure 1. Indicates the responses to the ‘Action taken’ scale at pre-training and 6 months post-training. [file 12888_2020_2805_MOESM1_ESM.docx]

Additional file 1 – Responses to ‘Action taken in response to suspected depression’ Scale

Supplementary Figure 1


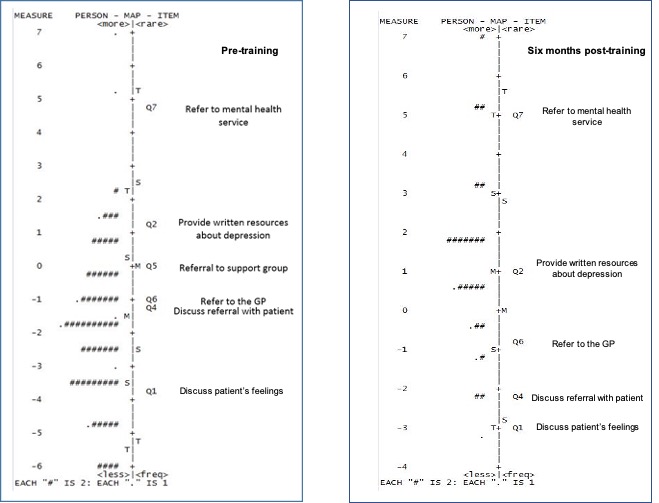


*Supplementary Figure 1: ‘Action taken’ scale responses at pre-training and six months post-training*

*Practitioners are represented on the left of the dashed line, with “#” equivalent to 2 people and “.” equivalent to 1 person. The items are represented on the right of the dashed line. The items at the top of the map represent actions that practitioners took rarely, while the items at the bottom are actions practitioners took more frequently.*
