# Supplementary material for: Barriers to integrating routine depression screening into community low vision rehabilitation services: a mixed methods study
Source: BMC Psychiatry. 2020 Aug 26;20:419. doi: 10.1186/s12888-020-02805-8 (PMC7448511; doi:10.1186/s12888-020-02805-8)
Supplement: Supplementary file 2 — Additional file 2: Responses to ‘Perceived barriers’ Scale. Supplementary Figure 2. Indicates the responses to the ‘Perceived barrier’ scale at pre-training and 6 months post-training. [file 12888_2020_2805_MOESM2_ESM.docx]

Additional file 2 – Responses to ‘Perceived barriers’ Scale

Supplementary Figure 2


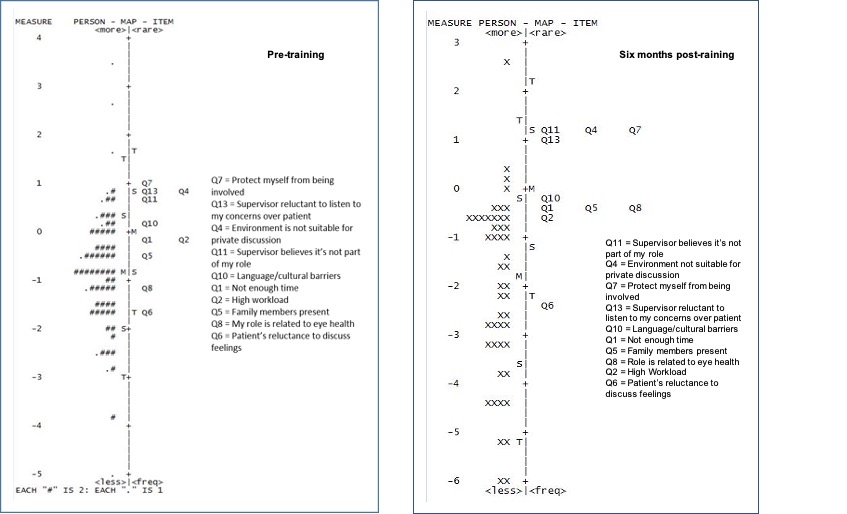


*Supplementary Figure 2: ‘Perceived barriers’ responses at pre-training and six months post-training*

*Practitioners are represented on the left of the dashed line, with “#” equivalent to 2 people and “.” or “x” equivalent to 1 person. The items are represented on the right of the dashed line. The items at the top of the map represent the barriers that practitioners reported least frequently, while items at the bottom were barriers that practitioners reported most frequently.*
